# Supplementary material for: Genome-wide CRISPR screen identifies ELP5 as a determinant of gemcitabine sensitivity in gallbladder cancer
Source: Nat Commun. 2019 Dec 2;10:5492. doi: 10.1038/s41467-019-13420-x (PMC6889377; doi:10.1038/s41467-019-13420-x)
Supplement: Supplementary file 1 — Supplementary Information [file 41467_2019_13420_MOESM1_ESM.pdf]

Supplementary Information for

Genome-wide CRISPR screen identifies ELP5 as a determinant of gemcitabine sensitivity in gallbladder cancer

Xu et al.

Supplementary Figures

Supplementary Fig. 1 Gemcitabine IC_{50} values in a panel of GBC cell lines and pathway analyses of 210 essential gene hits in the CRISPR screen.

Supplementary Fig. 2 ELP5 depletion is highly associated with GEM resistance.

Supplementary Fig. 3 ELP5 depletion did not affect GBC cell growth, but influenced GEM and cisplatin resistance.

Supplementary Fig. 4 U_{34} tRNA-modifying enzymes are required for GEM-induced cytotoxic effects, and U_{34} tRNA modification is activated under GEM treatment.

Supplementary Fig. 5 Lower ELP5 expression is associated with drug resistance and down-regulated P53 signatures.

Supplementary Fig. 6 IRES-dependent translation is inhibited in GBC cells depleted of U34 tRNA-modifying enzymes.

Supplementary Fig. 7 hnRNPQ regulates P53 translation in an IRES-dependent manner, and hnRNPQ expression is dependent on U_{34} tRNA-modifying enzymes.

Supplementary Fig. 8 U_{34}-independent hnRNPQ can resume P53 IRES activity and rescue GEM sensitivity in ELP5-depleted GBC cells.

Supplementary Fig. 9 The expression of U_{34} tRNA-modifying enzymes is negatively correlated with poor survival outcomes in GBC patients.

Supplementary Tables:

Supplementary Table 1. Codon counts of wobble U_{34} tRNA modification in the reported p53 ITAFs.

Supplementary Table 2. Clinical information on GBC patients received mini-PDX model test (Cohort 1).

Supplementary Table 3. Clinical information on GBC patients received gemcitabine-cisplatin therapy after surgery (Cohort 2).

Supplementary Table 4. Relationship between ELP5, hnRNPQ or P53 expression and clinicopathologic features of GBC patients (Cohort 2).

Supplementary Table 5: List primers used in this study**.**

Supplementary Fig. 1

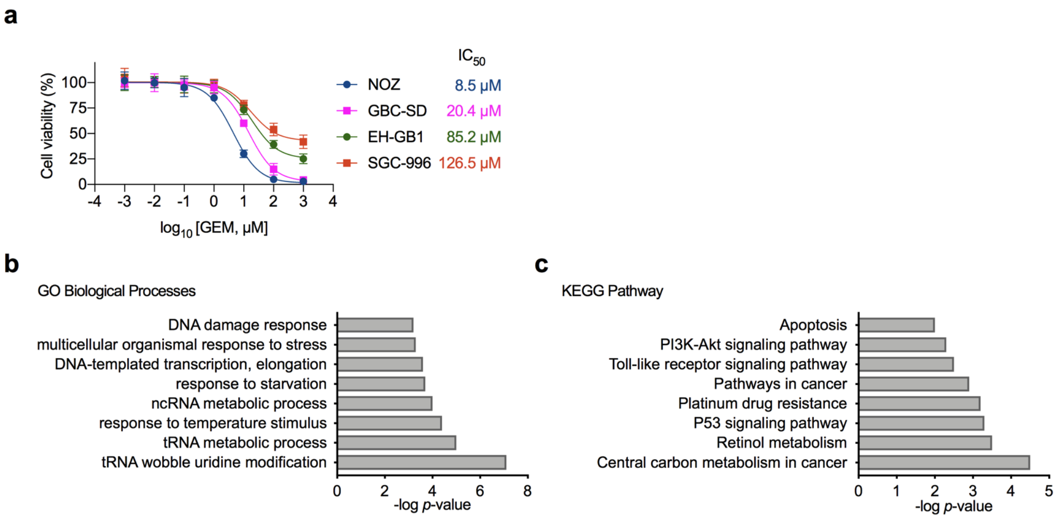

Supplementary Fig. 1 Gemcitabine IC_{50} values in a panel of GBC cell lines and pathway analyses of 210 essential gene hits in the CRISPR screen. **a** The NOZ cell line exhibited the highest sensitivity to gemcitabine (GEM) with the lowest IC_{50} value across our four GBC cell lines. Data represent the mean ± S.D., $n = 3$ independent experiments, error bars represent S.D. **b-c** Gene Ontology (GO) analysis (**b**) and Kyoto Encyclopedia of Genes and Genomes (KEGG) pathway analysis (**c**) showed that 210 essential gene hits mainly categorised in tRNA wobble uridine modification, metabolism, apoptosis, DNA damage, the P53 signalling pathway, and platinum drug resistance.

Supplementary Fig. 2

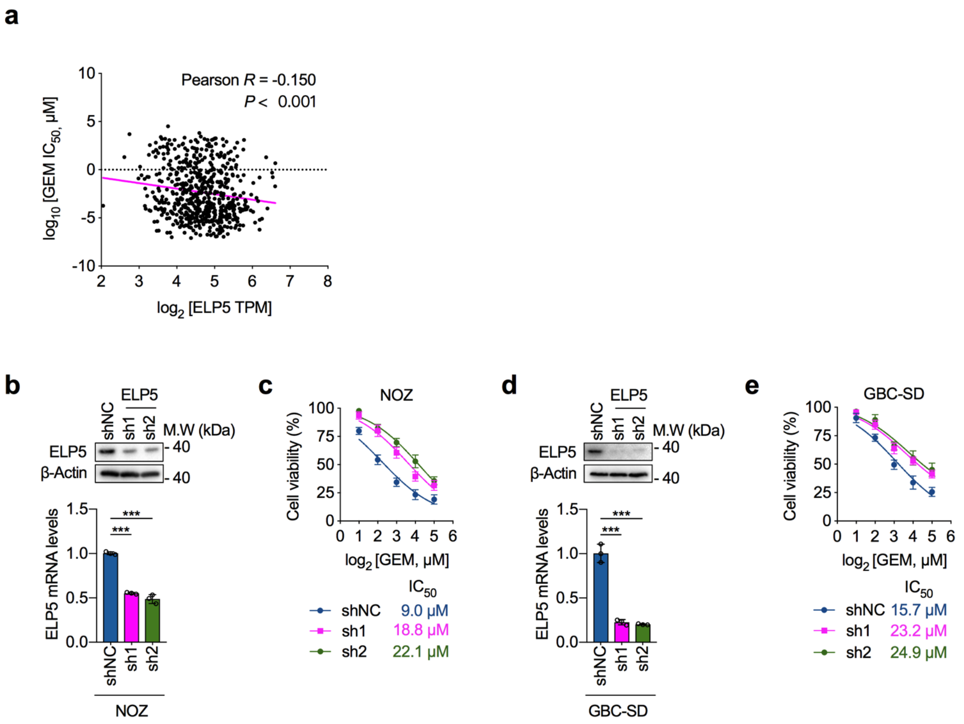

Supplementary Fig. 2 ELP5 depletion is highly associated with GEM resistance. **a** ELP5 mRNA levels across various cancer cell lines in the Cancer Cell Line Encyclopedia (CCLE) and Genomics of Drug Sensitivity in Cancer (GDSC) database were negatively correlated with GEM IC_{50}, as analysed by Pearson correlation coefficient. IC_{50}, 50% inhibitory concentration. TPM, transcripts per million. **b-e** ELP5 depletion in NOZ cells (**b**, **c**) and GBC-SD cells (**d**, **e**) by two independent shRNAs conferred GEM resistance and increased IC_{50}. shNC, non-specific control shRNA. Data represent the mean ± S.D., n = 3 independent experiments, in **b**, **c**, **d**, **e**, error bars represent S.D. Unpaired Student's t-tests were used in **b**, **d** ($^{***}P < 0.001$).

Supplementary Fig. 3

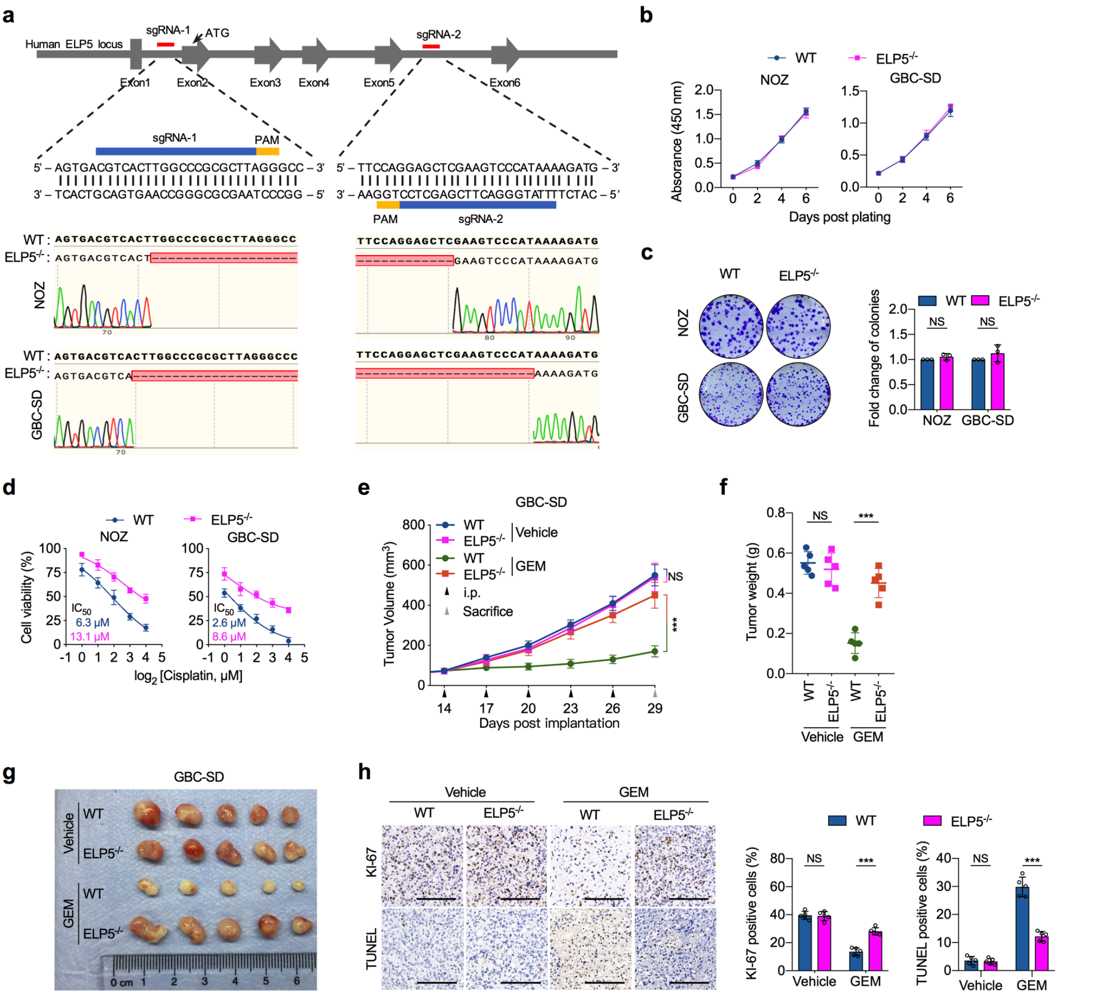

Supplementary Fig. 3 ELP5 depletion did not affect GBC cell growth, but influenced GEM and cisplatin resistance. **a** Schematic drawing of the method using two different sgRNAs targeting *ELP5* introns in the genome to delete exons containing the initiation codon with minor off-targets, as validated by genomic PCR sequencing. **b-c** ELP5 depletion was dispensable for GBC cell growth (**b**) and colony formation (**c**). **d** ELP5 depletion conferred resistance to cisplatin under cisplatin treatment for 72 h at the indicated doses. **e-h** ELP5 depletion prevented xenograft growth inhibition and apoptosis induced by GEM intraperitoneal injection (i.p.) in GBC-SD cell xenografts, which were assessed by tumour growth volume (**e**), tumour weight (**f**), representative images (**g**) of xenograft tumours after scarification, and KI-67 (upper) and TUNEL (lower) staining in paraffin-fixed xenograft tumour tissues after scarification (**h**); scale bars = 200 μm. 2×10^6 WT or ELP5$^{-/-}$ GBC-SD cells were injected subcutaneously into the right axilla of athymic nude mice (n = 5 animals per group). Data represent the mean ± S.D. in **b**, **c**, **d** (n = 3 independent experiments) and **e**, **f**, **h** (n = 5 per group), error bars represent S.D.. Unpaired Student's t-tests were used in **d**, **f**, **h**, and one-way ANOVA was used in **e** (NS, non-significant, $^{***}P < 0.001$).

Supplementary Fig. 4

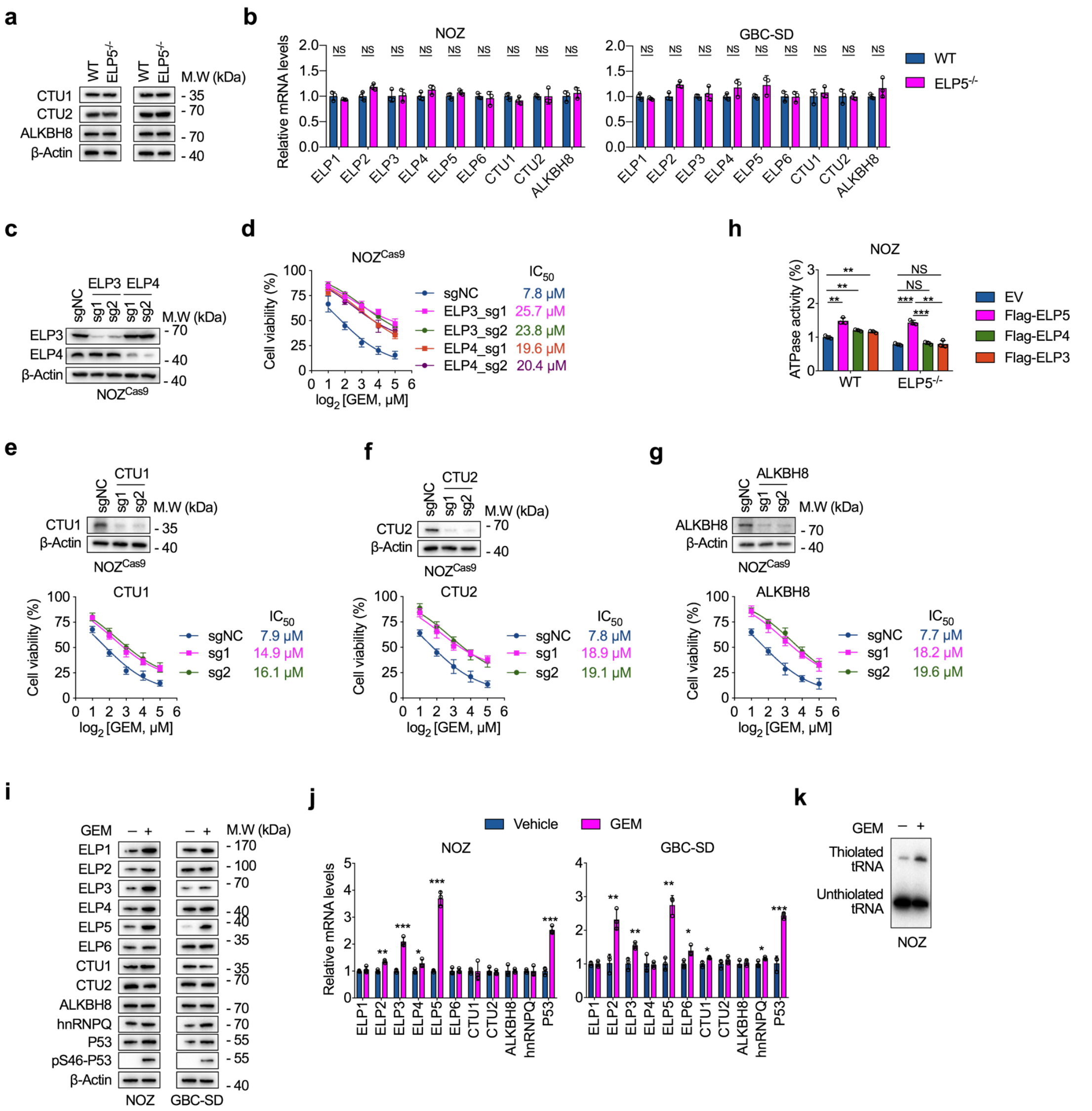

Supplementary Fig. 4 U_{34} tRNA-modifying enzymes are required for GEM-induced cytotoxic effects, and U_{34} tRNA modification is activated under GEM treatment. **a** ELP5 depletion did not affect the protein expression of other U_{34} tRNA-modifying enzymes, including CTU1, CTU2, and ALKBH8. **b** RT-qPCR analyses showed that ELP5 depletion did not affect the mRNA levels of Elongator subunits and other U_{34} tRNA-modifying enzymes. **c**, **d** *ELP3* and *ELP4* knockout via CRISPR/Cas9 in NOZ^{Cas9} cells (**c**) increased the IC_{50} of GEM (**d**). **e-g** *CTU1* (**e**), *CTU2* (**f**) and *ALKBH8* (**g**) knockout in NOZ^{Cas9} cells increased the IC_{50} of GEM. **h** Ectopically expressed ELP5 could respectively enhance or rescue ATPase activity in WT and $ELP5^{-/-}$ NOZ cells; ectopically expressed ELP3 or ELP4 could enhance ATPase activity in WT cells, but not in $ELP5^{-/-}$ cells. **i**, **j** Protein levels of all six Elongator subunits were increased under GEM treatment with IC_{50} for 72 h (**i**), but only partial transcripts were up-regulated (**j**). **k** The abundance of thiolated tE^{UUC} tRNA in NOZ cells was increased under GEM treatment with IC_{50} for 72 h. Data represent the mean ± S.D., n = 3 independent experiments in **b**, **d**, **e**, **f**, **g**, **h**, **j**, error bars represent S.D. Unpaired Student's t-tests were used in **b**, **h**, **j** (NS, non-significant, $^{*}P < 0.05$, $^{**}P < 0.01$ and $^{***}P < 0.001$).

Supplementary Fig. 5

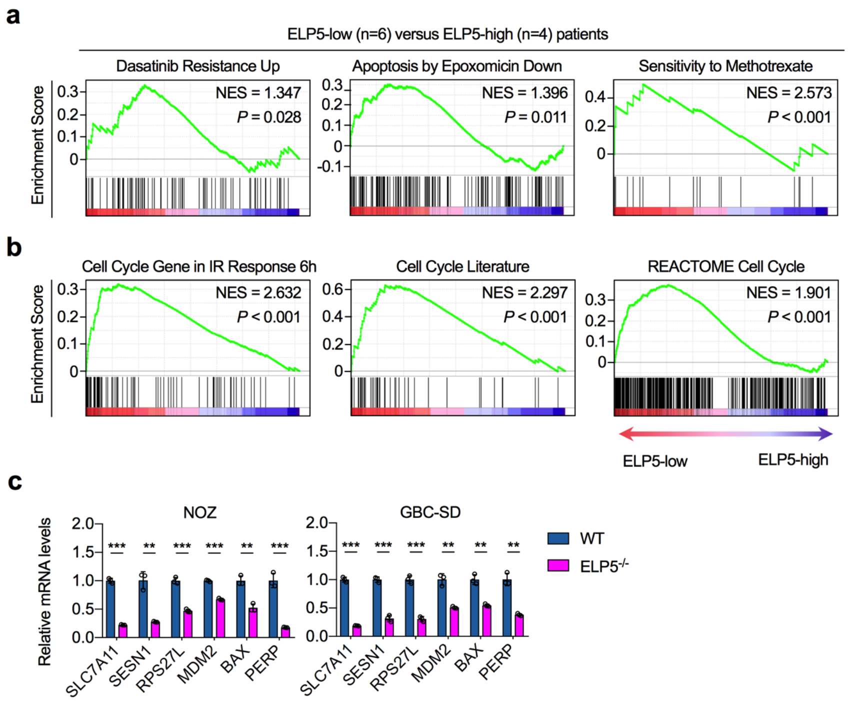

Supplementary Fig. 5 Lower ELP5 expression is associated with drug resistance and down-regulated P53 signatures. **a** Drug resistance-related biological signatures were enriched in GBC specimens with low ELP5 expression. **b** Cell cycle signatures were dysregulated in GBC specimens with low ELP5 expression. **c** RT-qPCR confirmed that ELP5 depletion in GBC cells down-regulated P53 target gene mRNA. Data in **c** represent the mean ± S.D., $n = 3$ independent experiments, error bars represent S.D., and unpaired Student's *t*-tests were used ($^{**}P < 0.001$ and $^{***}P < 0.001$).

Supplementary Fig. 6

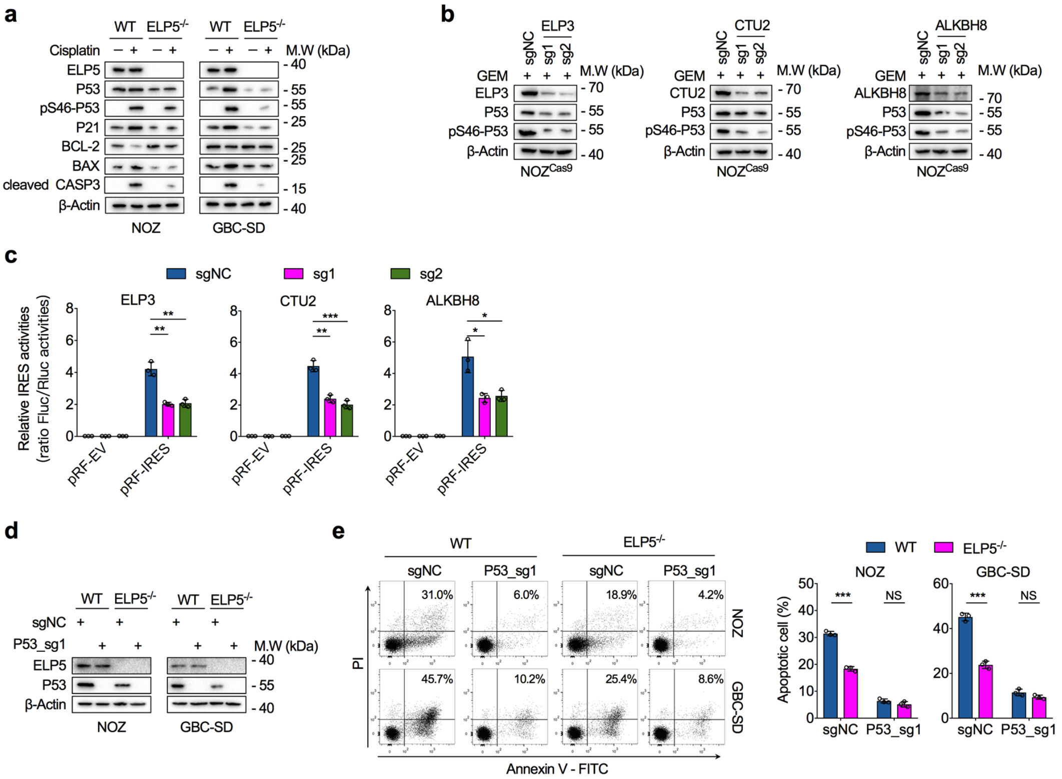

Supplementary Fig. 6 IRES-dependent translation is inhibited in GBC cells depleted of U34 tRNA-modifying enzymes. **a** ELP5 depletion substantially reduced the accumulation and activation of P53 and P53-mediated apoptosis under cisplatin treatment at IC_{50} for 72 h. **b** P53 accumulation and activation were reduced in NOZ^{Cas9} cells depleted of U_{34} tRNA-modifying enzymes. **c** *P53* IRES activity was significantly inhibited in NOZ^{Cas9} cells depleted of U_{34} tRNA-modifying enzymes. **d**, **e** *P53* knockout in *ELP5*-depleted GBC cells (**d**) could further reduce GEM-induced apoptosis (**e**). Data represent the mean ± S.D., $n = 3$ independent experiments in **c**, **e**, error bars represent S.D. Unpaired Student's *t*-tests were used in **c**, **e** (NS, non-significant, $^{*}P < 0.05$, $^{**}P < 0.01$ and $^{***}P < 0.001$).

Supplementary Fig. 7

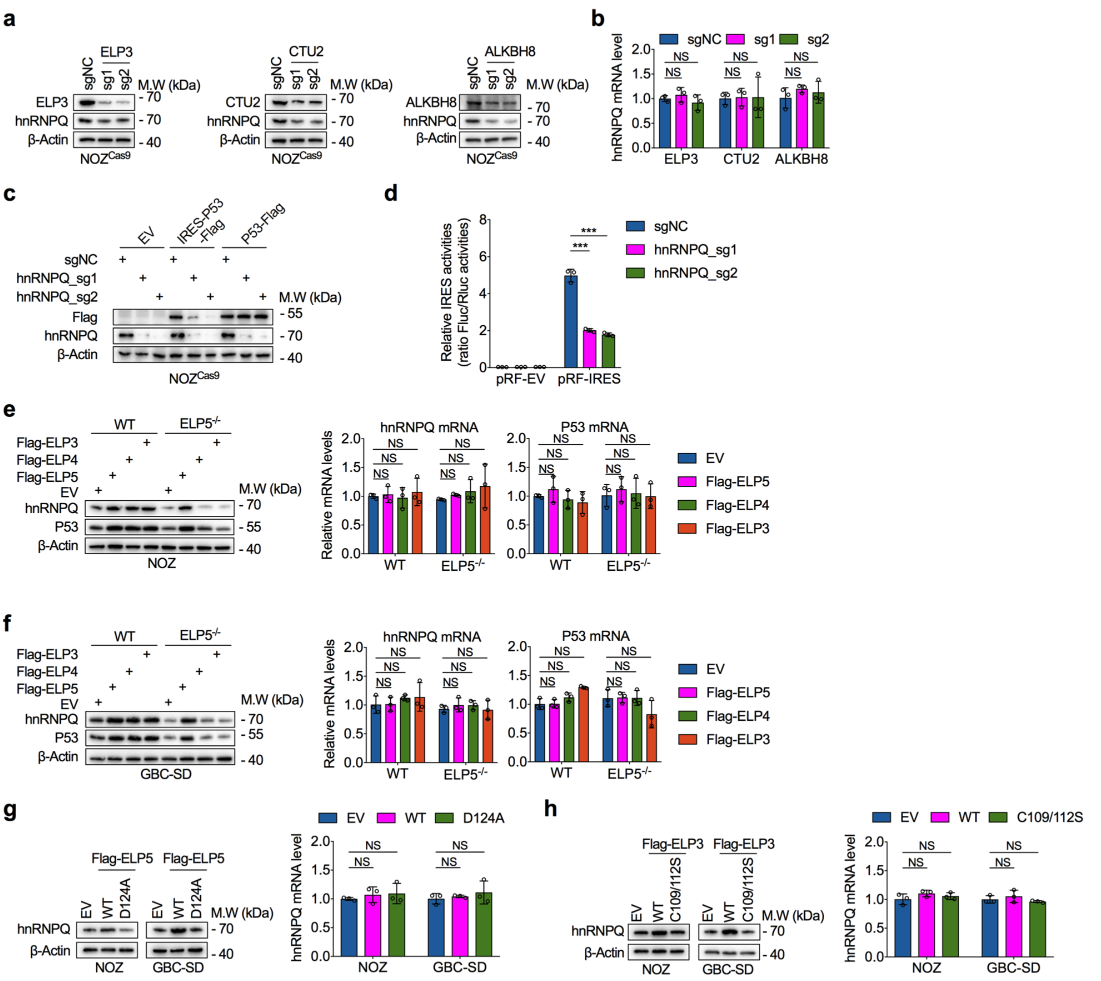

Supplementary Fig. 7 hnRNPQ regulates P53 translation in an IRES-dependent manner, and hnRNPQ expression is dependent on U_{34} tRNA-modifying enzymes. **a**, **b** *ELP3*, *CTU2*, and *ALKBH8* knockouts significantly affected the expression levels of hnRNPQ proteins (**a**), but not mRNA (**b**). **c**, **d** *P53* IRES-dependent translation (**c**) and IRES activity (**d**) were significantly inhibited in *hnRNPQ*-knockout cells. **e**, **f** Ectopically expressed ELP5 could promote and rescue the expression of hnRNPQ and P53 protein (**e**, **f** left panel), but not mRNA (**e**, **f** right panel), in WT and $ELP5^{-/-}$ cells, respectively; however, ectopically expressed ELP3 and ELP4 could only promote the expression of hnRNPQ and P53 protein (**e**, **f** left panel) in WT cells, not *ELP5*$^{-/-}$ cells. **g** Mutation of the ATPase activity site residue within ELP5 (D124A) could not promote the expression of hnRNPQ protein (left panel) and mRNA (right panel), but wild-type ELP5 could promote hnRNPQ mRNA translation. **h** Mutation of the cm^5U catalytic activity site residue within ELP3 (C109/112S) could not promote the expression of hnRNPQ protein (left panel) and mRNA (right panel), but wild-type ELP3 could promote hnRNPQ mRNA translation. Data represent the mean ± S.D., $n = 3$ independent experiments in **b**, **d**, **e**, **f**, **g**, **h**, error bars represent S.D. Unpaired Student's *t*-tests were used in **b**, **d**, **e**, **f**, **g**, **h** (NS, non-significant, $^{***}P < 0.001$).

Supplementary Fig. 8

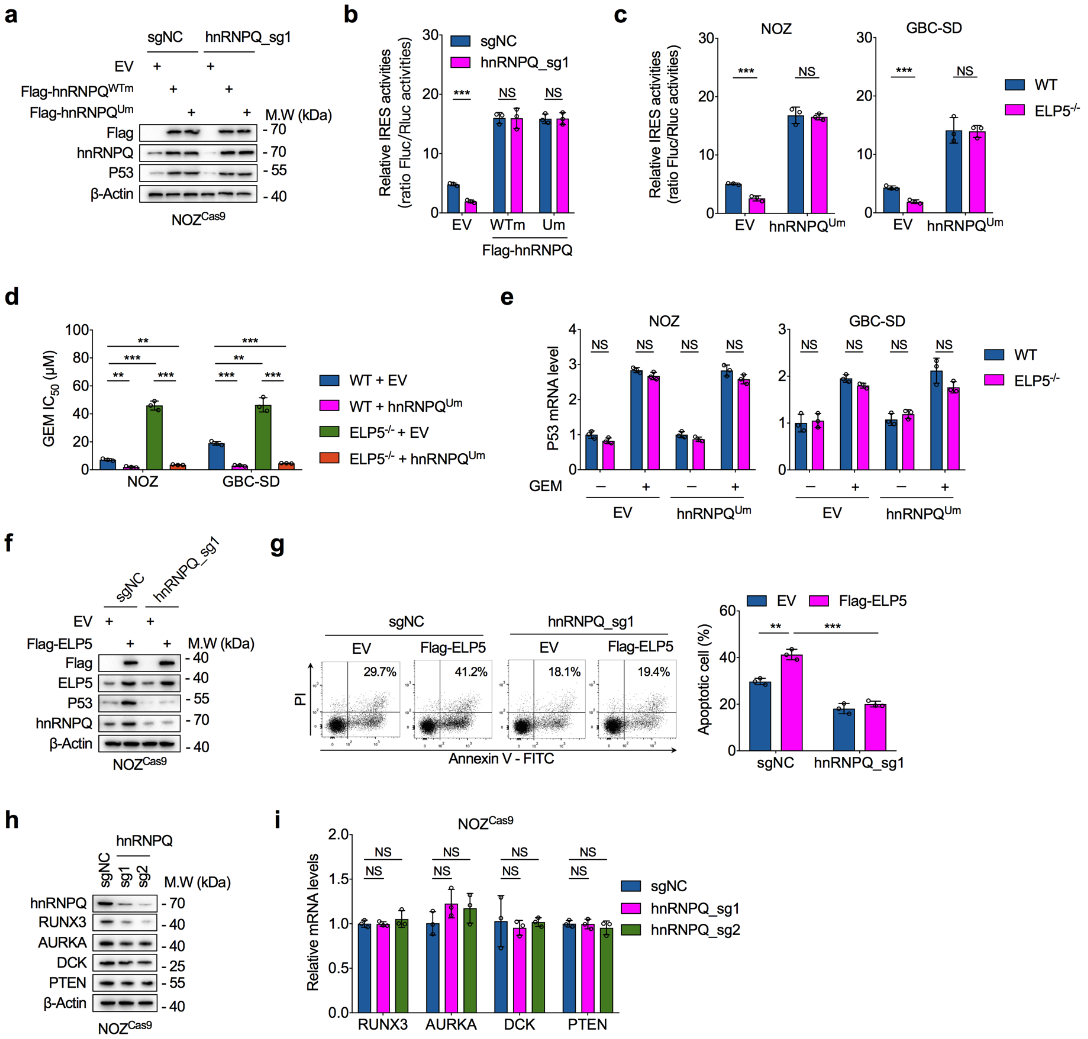

Supplementary Fig. 8 U_{34}-independent hnRNPQ can resume P53 IRES activity and rescue GEM sensitivity in ELP5-depleted GBC cells. **a**, **b** The hnRNPQ^{Um} rescue in *hnRNPQ*-knockout cells could promote P53 expression (**a**) and *P53* IRES activity (**b**) at levels comparable to those with hnRNPQ^{WTm} rescue. WTm, the hnRNPQ wild-type ORF sequence synonymously mutated at sgRNA targeting sites. **c** The hnRNPQ^{Um} rescue in *ELP5*-depleted GBC cells could restore and enhance *P53* IRES activity. **d** The IC_{50} of GEM with or without hnRNQP^{Um} overexpression in WT and *ELP5*$^{-/-}$ cells. See Fig. 7k. **e** The hnRNPQ^{Um} rescue could not affect *P53* transcription in WT and *ELP5*$^{-/-}$ cells. **f**, **g** Ectopically expressed ELP5 in *hnRNPQ*-knockout cells (**f**) could not rescue the GEM-induced apoptosis reduced by hnRNPQ depletion (**g**). **h**, **i** The *hnRNPQ* knockout reduced the translational expression of validated (i.e., AURKA) or potential targeted transcripts (i.e., *RUNX3*, *DCK*, *PTEN*) in protein (**h**), but not mRNA (**i**). Data represent the mean ± S.D., $n = 3$ independent experiments in **b**, **c**, **d**, **e**, **g**, **i**, error bars represent S.D. Unpaired Student's *t*-tests were used in **b**, **c**, **d**, **e**, **g**, **i** (NS, non-significant, $^{**}P < 0.01$ and $^{***}P < 0.001$).

Supplementary Fig. 9

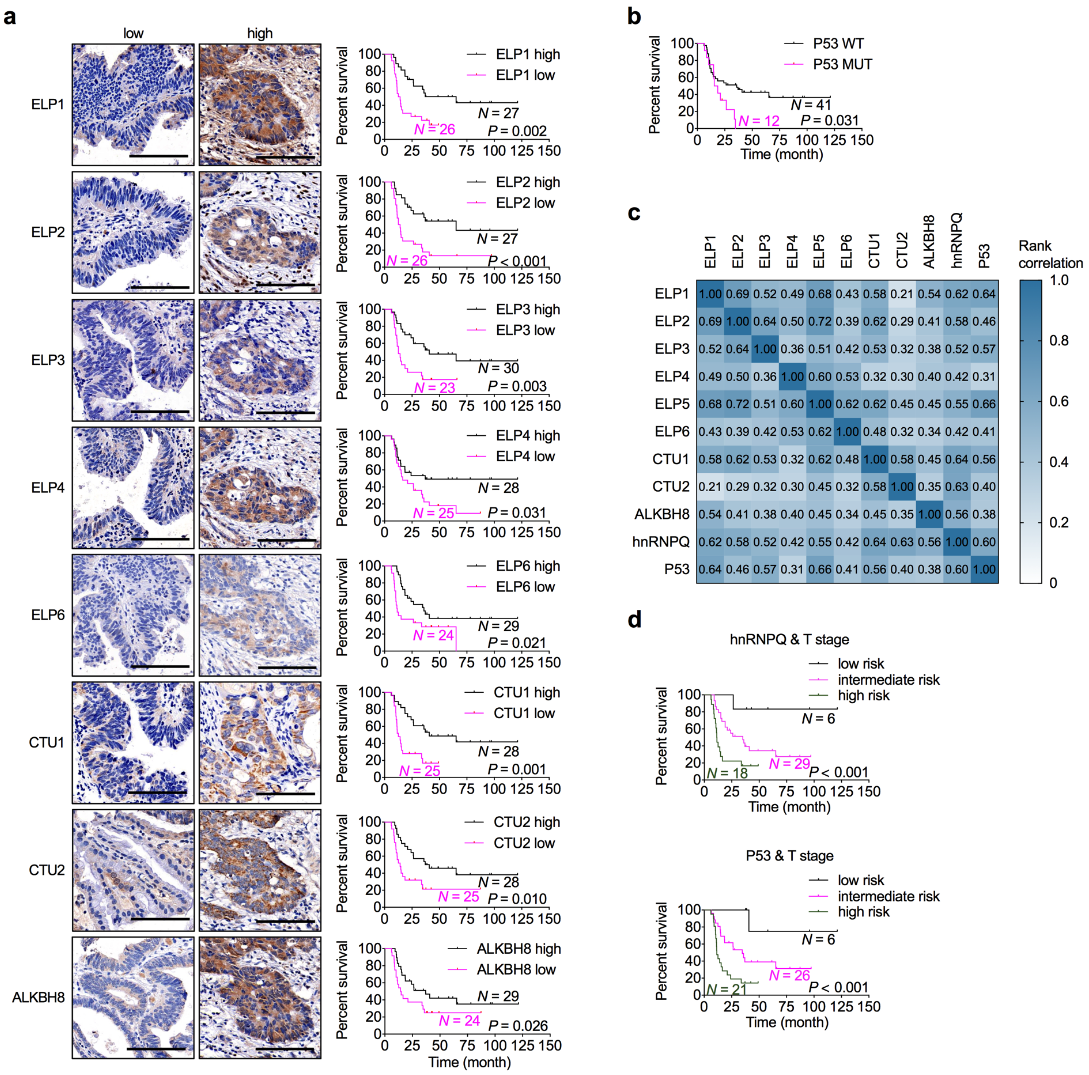

Supplementary Fig. 9 The expression of U_{34} tRNA-modifying enzymes is negatively correlated with poor survival outcomes in GBC patients. **a** Low expression levels of ELP1, ELP2, ELP3, ELP4, ELP6, CTU1, CTU2, or ALKBH8 were highly associated with poor overall survivals in GBC patients. Scale bars = 100 μm. **b** GBC patients with a mutated *P53* status exhibited poorer survival outcomes than patients with wild-type *P53* status. **c** The expression levels of all Elongator subunits, CTU1, CTU2, ALKBH8, hnRNPQ, and P53 in GBC tissues as assessed by histoscores were significantly positively correlated with each other. **d** Kaplan-Meier estimate of survival time in GBC patients who received gemcitabine-cisplatin therapy after surgery (Cohort 2) according to the combination of hnRNPQ or P53 and T stage. Log-rank tests were used in **a**, **b**, **d**; pearson correlation coefficients were used in **c**.

Supplementary Table 1. Codon counts of wobble U_{34} tRNA modification in the reported p53 ITAFs.

Protein	Codon counts	$mcm^5s^2U_{34}$			mcm^5U_{34}		ncm^5U_{34}						Total frequency
		CAA	AAA	GAA	AGA	GGA	UUA	GUA	UCA	CCA	ACA	GCA	
hnRNPQ	624	13	29	33	17	24	9	7	5	15	10	16	0.285
DAP5	907	907	24	39	47	11	28	17	10	9	25	9	0.262
PSF	707	14	21	42	16	22	2	2	2	30	6	11	0.238
RHA	1270	26	35	61	25	47	8	21	10	23	14	27	0.234
RPL26	145	2	11	8	1	2	0	4	0	0	1	1	0.207
ANXA2	357	2	11	10	8	6	1	1	2	1	4	6	0.146
TCP80	898	9	22	17	11	17	2	5	9	20	8	10	0.145
hnRNPL	589	5	9	9	3	12	1	3	3	14	2	7	0.115

Supplementary Table 2. Clinical information on GBC patients received mini-PDX model test (Cohort 1).

NO.	Pathological differentiation	AJCC stage[a]	Gemcitabine viability (%)	Histoscore		
				ELP5	hnRNPQ	P53
1	Poor	IIIb	130	0	0	0
2	Poor	IIIb	55	0	0	2
3	Moderate	IIIb	89	0	0	2
4	Poor	IIIa	71	3	0	0
5	Poor	IIIa	138	2	1	0
6	Poor	IVb	68	0	6	4
7	Poor	IVb	85	2	6	1
8	Moderate	IIIb	59	3	9	1
9	Poor	IVa	14	12	4	8
10	Poor	IIIb	-24	8	8	9
11	Moderate	IVb	26	6	9	12
12	Poor	IVb	42	4	6	9
13	Moderate	IIIa	-116	8	12	9
14	Poor	IVb	56	4	6	0
15	Moderate	IVa	77	6	3	4
16	Moderate	IVb	22	9	12	4

[a]AJCC stage, based on the American Joint Committee in Cancer (AJCC)/International Union Against Cancer staging manual (8th edition).

Supplementary Table 3. Clinical information on GBC patients received gemcitabine-cisplatin therapy after surgery (Cohort 2).

NO.	Pathological differentiation	T stage	AJCC stage[a]	Histoscore											P53 genotype
				ELP5	hnRNPQ	P53	ELP1	ELP2	ELP3	ELP4	ELP6	CTU1	CTU2	ALKBH8	
1	Moderate	3	IIIa	6	1	4	3	3	8	6	8	8	1	2	MUT (H168 framshift)
2	Moderate	2a	IIa	6	6	6	6	6	9	8	8	6	4	8	WT
3	Poor	3	IIIa	8	3	3	2	8	9	3	3	3	3	6	WT
4	Moderate	2b	IIb	2	2	2	4	1	1	0	4	2	3	3	MUT (D281G)
5	Well	4	IVa	9	12	8	9	12	12	3	2	12	8	8	WT
6	Moderate	3	IIIb	1	8	2	8	6	6	8	1	1	2	8	MUT (P190L)
7	Well	1a	I	6	2	4	2	2	8	3	8	6	6	6	WT
8	Poor	3	IIIb	9	6	8	8	8	8	2	9	6	0	2	WT
9	Moderate	3	IIIa	6	6	8	6	8	9	9	8	3	0	6	MUT (D281H)
10	Poor	3	IIIa	6	9	9	9	3	9	6	9	9	12	8	WT
11	Moderate	4	IVa	2	2	0	2	4	1	4	1	6	1	1	WT
12	Moderate	4	Iva	4	8	2	4	4	3	3	8	8	4	1	MUT (S127P)
13	Poor	3	IIIa	2	2	3	4	1	0	2	0	2	2	1	WT
14	Moderate	3	IIIa	2	6	2	2	3	6	6	3	2	6	1	WT
15	Moderate	4	IVa	3	2	2	2	3	3	3	3	4	2	2	WT
16	Moderate	4	IVb	6	4	8	3	4	6	2	0	6	6	6	WT
17	Poor	3	IIIb	12	8	8	9	12	12	12	9	6	6	6	WT
18	Poor	3	IIIb	12	8	8	3	1	3	12	9	3	12	6	WT
19	Poor	3	IIIa	2	3	2	2	2	6	2	6	2	2	3	WT
20	Poor	3	IIIa	3	3	2	4	2	3	3	2	2	2	0	WT
21	Moderate	3	IIIa	4	6	8	6	0	3	4	4	6	4	6	WT
22	Moderate	3	IIIa	1	2	2	2	1	3	4	6	2	1	4	WT
23	Poor	4	IVa	1	4	4	1	0	1	1	1	1	4	1	WT
24	Poor	3	IIIa	6	6	8	8	6	8	2	8	12	6	3	WT
25	Poor	3	IIIb	1	8	8	8	1	8	1	1	1	1	6	MUT (Y205S)
26	Moderate	3	IIIb	1	3	0	1	2	2	2	6	1	1	1	WT
27	Moderate	3	IIIa	4	6	4	4	4	9	9	6	4	6	6	WT
28	Moderate	3	IIIa	6	6	0	3	6	0	1	6	2	6	6	MUT (R248 stopped)

29	Well	1b	I	8	4	8	8	8	12	6	8	8	4	8	WT
30	Poor	3	IIIa	12	8	6	12	9	2	12	8	8	2	12	MUT (R280K)
31	Poor	3	IIIa	3	3	3	3	3	3	3	9	2	4	6	WT
32	Moderate	1a	I	4	4	4	6	4	6	4	2	6	4	6	WT
33	Moderate	4	IVb	0	0	0	1	1	1	0	0	1	2	2	WT
34	Moderate	3	IIIa	4	12	3	2	6	8	8	8	12	12	8	WT
35	Moderate	3	IIIb	0	2	2	2	0	0	4	2	0	2	2	WT
36	Poor	3	IIIa	12	9	12	9	9	12	9	8	12	9	4	WT
37	Moderate	3	IIIa	2	2	2	2	4	6	1	1	2	8	0	WT
38	Poor	3	IIIa	4	6	12	4	4	9	3	6	4	4	4	WT
39	Moderate	3	IIIa	0	0	0	0	1	0	0	0	1	2	2	MUT (M160V)
40	Moderate	2a	IIa	12	8	8	9	12	8	12	9	12	9	2	WT
41	Well	1a	I	2	3	0	2	2	4	2	2	3	3	6	WT
42	Moderate	2a	IIa	1	4	2	1	4	2	4	1	4	6	2	WT
43	Poor	3	IIIb	1	0	0	1	1	3	6	1	1	0	2	WT
44	Poor	3	IIIa	1	3	4	1	2	8	4	3	1	3	2	MUT (P47Q)
45	Moderate	3	IIIa	4	4	6	4	4	8	6	6	8	4	6	WT
46	Poor	4	IVa	8	6	8	6	3	2	6	6	8	8	8	WT
47	Poor	2a	IIIb	8	3	4	8	8	9	6	3	3	2	3	WT
48	Poor	4	IVa	2	6	0	6	8	8	8	6	8	6	8	WT
49	Poor	4	IVa	1	0	0	1	0	0	0	2	1	2	2	WT
50	Poor	3	IIIb	6	9	12	6	9	6	2	2	12	8	8	WT
51	Poor	3	IIIb	6	2	4	8	6	3	9	6	6	3	6	WT
52	Moderate	2b	IIb	3	2	0	2	3	3	9	3	0	3	3	MUT (R248P)
53	Well	2b	IIb	4	6	0	4	3	8	2	4	9	6	8	MUT (Q317E)

[a]AJCC stage, based on the American Joint Committee in Cancer (AJCC)/International Union Against Cancer staging manual (8th edition).

Supplementary Table 4. Relationship between ELP5, hnRNPQ or P53 expression and clinicopathologic features of GBC patients (Cohort 2).

Features	Relative ELP5 expression		*P* value	Relative hnRNPQ expression		*P* value	Relative P53 expression		*P* value
	Low	High		Low	High		Low	High	
Case	24	29		23	30		26	27	
Gender									
Male	12	8	0.154	11	9	0.255	12	8	0.264
Female	12	21		12	21		14	19	
Age									
≤ 65 y	11	21	0.089	12	20	0.397	13	19	0.166
> 65 y	13	8		11	10		13	8	
Pathological differentiation									
Well & Moderate	14	16	1.000	13	17	1.000	18	12	0.098
Poor	10	13		10	13		8	15	
Tumor size									
≤ 3 cm	14	15	0.783	12	17	0.786	17	12	0.170
> 3 cm	10	14		11	13		9	15	
T stage									
$T_{1\sim2}$	4	7	0.735	5	6	1.000	5	6	1.000
$T_{3\sim4}$	20	22		18	24		21	21	
N stage									
N_0	14	19	0.776	14	19	1.000	16	17	1.000
$N_{1\sim2}$	10	10		9	11		10	10	
Liver metastasis									
Negative	13	20	0.394	15	18	0.799	16	17	1.000
Positive	11	9		8	12		10	10	
AJCC TNM stage									
Ⅰ~Ⅱ	4	6	1.000	4	6	1.000	5	5	1.000
Ⅲ~Ⅳ	20	23		19	24		21	21	

[a]AJCC stage, based on the American Joint Committee in Cancer (AJCC)/International Union Against Cancer staging manual (8th edition).

Supplementary Table 5: List primers used in this study

Real time PCR primer	Forward (5'-3')	Reverse (5'-3')
β-Actin	CATGTACGTTGCTATCCAGGC	CTCCTTAATGTCACGCACGAT
ELP5	AGCGAGGAAGAGTTTCGTGA	GGAAAGGCCTCCTCAGTTTT
ELP4	ACTGCATTTGCCTCCAGACT	GAAGTCCAGGTGCTTCTTGC
ELP3	CGTGCCAGATATGACCCTTT	CCATAAACGTTCCACCCATC
P53	GAGAGCTGAATGAGGCCTTG	TTATGGCGGGAGGTAGACTG
P21	CCATGTGGACCTGTCACTGT	GGCGTTTGGAGTGGTAGAAA
MDM2	ATGCCATTGAACCTTGTGTG	GGCAGGGCTTATTCCTTTTC
hnRNPQ	GGCAAGGCGTAGGTTAATGA	CAAGGTTGCGTACAAACAGC
SLC7A11	CCCTCTATTCGGACCCATTT	CCTGGGTTTCTTGTCCCATA
SESN1	ATTCGGCTGTGGAATCAGTC	TCCACACTGTGATTGCCATT
RPS27L	AGATCACCACGGTTTTCAGC	TCTGTGAGTCTGGCCTTTCC
BAX	GGGGACGAACTGGACAGTAA	CAGTTGAAGTTGCCGTCAGA
PERP	TTCCAGATCATCTCCCTGGT	AAAGCCGTAGGCCCAGTTAT
ELP1	GGAGCGTGGGAGATAATTCA	CAGTTGGTAGGTGCACATGG
ELP2	GTGCTCTATGACCCCCTGAA	GGGAGCCATCCTGTTTACAA
ELP6	GTGGCACTCATCCAGTCCTT	GACGTCCACTGCAGACTTGA
CTU1	GCACCTTCTGTGGAGTGCT	AGTTCATGAGCACGGTCTCC
CTU2	TGTGTGAAGTGCAAGGAAGC	CATGGCTCTGAACTTGTGGA
ALKBH8	TGGTTGTGATCGTAGCCAAA	GAGATGCAGGCATCACAAGA
RUNX3	GACCATCACTGTGTTCACCAA	GTCTGGTCCTCCAGCTTCTG
AURKA	CCAGGGACCTCATTTCAAGA	TTGGCAATTTGATGGTTTTG
DCK	GCCACTCCAGAGACATGCTT	CTATGCAGGAGCCAGCTTTC
PTEN	AGACCATAACCCACCACAGC	TTCGTCCCTTTCCAGCTTTA
**sgRNA sequence**	**Sense (5'-3')**	**Antisense (5'-3')**
sgP53_1	CCATTGTTCAATATCGTCCG	CGGACGATATTGAACAATGG
sgP53_2	GAGCGCTGCTCAGATAGCGA	TCGCTATCTGAGCAGCGCTC
sgP53_3	GGTGCCCTATGAGCCGCCTG	CAGGCGGCTCATAGGGCACC
sgP53_4	GATCCACTCACAGTTTCCAT	ATGGAAACTGTGAGTGGATC
sgELP5_1	CACACTCACTTTCCCCACTG	CAGTGGGGAAAGTGAGTGTG

sgELP5_2	ATGCAGGACCTGGCAGAGTG	CACTCTGCCAGGTCCTGCAT
sgELP5_3	AGGATCTGTCCTCTTGCACA	TGTGCAAGAGGACAGATCCT
sgELP5_4	GGGCCTCCGACACAGGATGT	ACATCCTGTGTCGGAGGCCC
sghnRNPQ_1	TATTCCTAAGAGTAAAACCA	TGGTTTTACTCTTAGGAATA
sghnRNPQ_2	GATGACAAGAAAAAAAACAG	GATGACAAGAAAAAAAACAG
sgELP3_1	TCCGAGCCACCAATTCAACC	GGTTGAATTGGTGGCTCGGA
sgELP3_2	TATTCCACCCAGTCTTACAC	GTGTAAGACTGGGTGGAATA
sgELP4_1	GCCAGCGTGACCAACGACAG	CTGTCGTTGGTCACGCTGGC
sgELP4_2	CCAACGGCTAAACCTCCACC	GGTGGAGGTTTAGCCGTTGG
sgCTU1_1	GTGCTCATGAACTTCCTACG	CGTAGGAAGTTCATGAGCAC
sgCTU1_2	GGGCTGGACGATGGACGCCG	CGGCGTCCATCGTCCAGCCC
sgCTU2_1	TCCGGCTCGTATCACCACAA	TTGTGGTGATACGAGCCGGA
sgCTU2_2	GCAGAGCACCAAAGCACCGA	TCGGTGCTTTGGTGCTCTGC
sgALKBH8_1	ATCACCAGCAAACTCCGACG	CGTCGGAGTTTGCTGGTGAT
sgALKBH8_2	TCAGCCACTATTGAACCACT	AGTGGTTCAATAGTGGCTGA
ELP5 exon deletion sgRNA	Sense (5'-3')	Antisense (5'-3')
sgRNA-1	CGTCACTTGGCCCGCGCTTA	TAAGCGCGGGCCAAGTGACG
sgRNA-2	TTTATGGGACTTCGAGCTCC	GGAGCTCGAAGTCCCATAAA
shRNA	Sense (5'-3')	Antisense (5'-3')
shELP5_1	CTGGTTTACCATGACTTCT	AGAAGTCATGGTAAACCAG
shELP5_2	CACAACTCATTTGACCTTT	AAAGGTCAAATGAGTTGTG
PCR primer for gRNA library		
lentiGP-F	5'-AATGGACTATCATATGCTTACCGTAACTTGAAAGTATTTCG-3'	
lentiGP-R	5'-CTTTAGTTTGTATGTCTGTTGCTATTATGTCTACTATTCTTTCC-3'	
Northern Blot probe		
tE^{UUC}	5′-TTCCCATACCGGGAGTCGAACCCG-3′	
