# Supplementary material for: Genome-wide CRISPR screen identifies ELP5 as a determinant of gemcitabine sensitivity in gallbladder cancer
Source: Nat Commun. 2019 Dec 2;10:5492. doi: 10.1038/s41467-019-13420-x (PMC6889377; doi:10.1038/s41467-019-13420-x)
Supplement: Supplementary file 3 — Description of Additional Supplementary Files [file 41467_2019_13420_MOESM3_ESM.pdf]

Description of Additional Supplementary Files

File Name: Supplementary Data 1
Description: Excel file contains the list of 210 essential gene hits in pooled screen using MAGeCK method (Tab 1), Gene Ontology (GO) biological processes analysis for gene hits (Tab 2), Kyoto Encyclopedia of Genes and Genomes (KEGG) pathway analysis for gene hits (Tab 3), and reads out counts (Tab 4).

File Name: Supplementary Data 2
Description: Excel file contains Gene Set Enrichment Analysis (GSEA) of GBC transcriptional profile between low and high ELP5 expression (Tab 1), RPKM files (Tab 2).
