# Supplementary material for: Genome-wide CRISPR screen identifies ELP5 as a determinant of gemcitabine sensitivity in gallbladder cancer
Source: Nat Commun. 2019 Dec 2;10:5492. doi: 10.1038/s41467-019-13420-x (PMC6889377; doi:10.1038/s41467-019-13420-x)
Supplement: Supplementary file 6 — Reporting Summary [file 41467_2019_13420_MOESM6_ESM.pdf]

nature research

Corresponding author(s): DBPR, NCOMMS-19-03138B

Last updated by author(s): Nov 1, 2019

Reporting Summary

Nature Research wishes to improve the reproducibility of the work that we publish. This form provides structure for consistency and transparency in reporting. For further information on Nature Research policies, see Authors & Referees and the Editorial Policy Checklist .

Statistics

For all statistical analyses, confirm that the following items are present in the figure legend, table legend, main text, or Methods section.

n/a	Confirmed	
☐	☒	The exact sample size (*n*) for each experimental group/condition, given as a discrete number and unit of measurement
☐	☒	A statement on whether measurements were taken from distinct samples or whether the same sample was measured repeatedly
☐	☒	The statistical test(s) used AND whether they are one- or two-sided<br>*Only common tests should be described solely by name; describe more complex techniques in the Methods section.*
☐	☒	A description of all covariates tested
☐	☒	A description of any assumptions or corrections, such as tests of normality and adjustment for multiple comparisons
☐	☒	A full description of the statistical parameters including central tendency (e.g. means) or other basic estimates (e.g. regression coefficient) AND variation (e.g. standard deviation) or associated estimates of uncertainty (e.g. confidence intervals)
☐	☒	For null hypothesis testing, the test statistic (e.g. *F*, *t*, *r*) with confidence intervals, effect sizes, degrees of freedom and *P* value noted<br>*Give P values as exact values whenever suitable.*
☒	☐	For Bayesian analysis, information on the choice of priors and Markov chain Monte Carlo settings
☒	☐	For hierarchical and complex designs, identification of the appropriate level for tests and full reporting of outcomes
☐	☒	Estimates of effect sizes (e.g. Cohen's *d*, Pearson's *r*), indicating how they were calculated

Our web collection on statistics for biologists contains articles on many of the points above.

Software and code

Policy information about availability of computer code

Data collection	Illumina HiSeq2500.
Data analysis	Gene Set Enrichment Analysis (GSEA), Microsoft Excel 2016, Graphpad Prism 7, Image J, Flow Jo, Photoshop, SPSS software, MAGeCk.

For manuscripts utilizing custom algorithms or software that are central to the research but not yet described in published literature, software must be made available to editors/reviewers. We strongly encourage code deposition in a community repository (e.g. GitHub). See the Nature Research guidelines for submitting code & software for further information.

Data

Policy information about availability of data

All manuscripts must include a data availability statement. This statement should provide the following information, where applicable:

- Accession codes, unique identifiers, or web links for publicly available datasets
- A list of figures that have associated raw data
- A description of any restrictions on data availability

The accession number for the transcriptional profiles is GSE139682.
Unprocessed gel bot of Fig. 1b, d, 2a, d, 3a, c–g, i, k, 5a, c, e, h, 6b, d, g, h, j and m, Supplementary Fig. 2b, d, 4a, c, e–g, i, k, 6a, b, d, 7a, c, e–g, h, 8a, f and i are provided in Source Data file.
The source data underlying Fig.1c, g, 2b, c, e, f, h, i, k, 3b, h, j, l, 5b, c, g, i, 6c, e, g, h, k and l, Supplementary Fig. 1a, 2a, c, e, 3b–f, h, 4b, d, e–h, j, 5c, 6c, e, 7b, d, e –h, 8b–e, g and j are provided in Source Data file

Field-specific reporting

Please select the one below that is the best fit for your research. If you are not sure, read the appropriate sections before making your selection.

- [x] Life sciences
- [ ] Behavioural & social sciences
- [ ] Ecological, evolutionary & environmental sciences

For a reference copy of the document with all sections, see nature.com/documents/nr-reporting-summary-flat.pdf

Life sciences study design

All studies must disclose on these points even when the disclosure is negative.

Sample size	The sample size of GBC Cohort 1 and GBC Cohort 2 was determined by the number of tumor samples and clinic information availabled in our database. Sample size and number of independent experiments are always clearly stated in the figure legend or in the Methods section. In CRISPR screen, two independent results were analyzed. In other experiments, three or more independent results were used to perform statistical analyses. All raw data required for statistical tests are indicated in Source Data file 2.
Data exclusions	No data were excluded from analysis.
Replication	Experiments in the article are reliably produced, replication were described in the figure legends.
Randomization	Animals with similar age and weight were randomly allocated to experimental groups.
Blinding	Investigators were not blinded to group allocation during data collection and/or analysis.

Reporting for specific materials, systems and methods

We require information from authors about some types of materials, experimental systems and methods used in many studies. Here, indicate whether each material, system or method listed is relevant to your study. If you are not sure if a list item applies to your research, read the appropriate section before selecting a response.

Materials & experimental systems

n/a	Involved in the study
[ ]	[x] Antibodies
[ ]	[x] Eukaryotic cell lines
[x]	[ ] Palaeontology
[ ]	[x] Animals and other organisms
[ ]	[x] Human research participants
[x]	[ ] Clinical data

Methods

n/a	Involved in the study
[x]	[ ] ChIP-seq
[ ]	[x] Flow cytometry
[x]	[ ] MRI-based neuroimaging

Antibodies

Antibodies used

For WB:
Mouse anti-ELP5 antibody (dilution: 1:100, Supplier: Santa Cruz, Cat:sc-514018)
Rabbit anti-ELP1 antibody (dilution: 1:5000, Supplier: Abcam, Cat: ab179437)
Rabbit anti-ELP2 antibody (dilution: 1:1000, Supplier: Abcam, Cat: ab154643)
Rabbit anti-ELP3 antibody (dilution: 1:3000, Supplier: Abcam, Cat: ab190907)
Rabbit anti-ELP4 antibody (dilution: 1:1000, Supplier: Abcam, Cat: ab133687)
Rabbit anti-CTU1 antibody (dilution: 1:500, Supplier: Abcam, Cat: ab136083)
Rabbit anti-CTU2 antibody (dilution: 1:1000, Supplier: Abcam, Cat: ab177160)
Rabbit anti-cleaved CASP-3 antibody (dilution: 1:500, Supplier: Abcam, Cat: ab32042)
Rabbit anti-RUNX3 antibody (dilution: 1:1000, Supplier: Abcam, Cat: ab224641)
Rabbit anti-AURKA antibody (dilution: 1:10000, Supplier: Abcam, Cat: ab52973)
Rabbit anti-PTEN antibody (dilution: 1:1000, Supplier: Abcam, Cat: ab32199)
Rabbit anti-ELP6 antibody (dilution: 1:1000, Supplier: NOVUS, Cat: NBP1-91733)
Mouse anti-P53 antibody (dilution: 1:1000, Supplier: Cell Signaling Technology, Cat: #2524)
Rabbit anti-pSer46-P53 antibody (dilution: 1:1000, Supplier: Cell Signaling Technology, Cat: #2521)
Rabbit anti-P21 antibody (dilution: 1:1000, Supplier: Cell Signaling Technology, Cat: #2947)
Rabbit anti-hnRNPQ antibody (dilution: 1:1000, Supplier: Abclonal, Cat: A7219)
Rabbit anti-ALKBH8 antibody (dilution: 1:1000, Supplier: Abclonal, Cat: A7142)
Rabbit anti-DCK antibody (dilution: 1:3000, Supplier: Abclonal, Cat: A1794)
Rabbit anti-BAX antibody (dilution: 1:2000, Supplier: Proteintech, Cat: 50599-2-Ig)

Rabbit anti-BCL-2 antibody (dilution: 1:1000, Supplier: Proteintech, Cat: 12789-1-AP)
Rabbit anti-DAP5 antibody (dilution: 1:500, Supplier: Proteintech, Cat: 17728-1-AP)
Rabbit anti-PSF antibody (dilution: 1:1000, Supplier: Proteintech, Cat: 15585-1-AP)
Rabbit anti-RHA antibody (dilution: 1:1000, Supplier: Proteintech, Cat: 17721-1-AP)
Rabbit anti-RPL26 antibody (dilution: 1:1000, Supplier: Proteintech, Cat: 17619-1-AP)
Rabbit anti-ANXA2 antibody (dilution: 1:3000, Supplier: Proteintech, Cat: 11256-1-AP)
Rabbit anti-TCP80 antibody (dilution: 1:1000, Supplier: Proteintech, Cat: 19887-1-AP)
Rabbit anti-hnRNPL antibody (dilution: 1:500, Supplier: Proteintech, Cat: 18354-1-AP)
Rabbit anti-Flag antibody (dilution: 1:10000, Supplier: Sigma-Aldrich, Cat: SAB1306078)
Mouse anti-β-actin antibody (dilution: 1:10000, Supplier: Sigma-Aldrich, Cat: A1978)

For IHC:
Rabbit anti-ELP5 antibody (dilution: 1:200, Supplier: Sigma-Aldrich, Cat: HPA023279)
Rabbit anti-hnRNPQ antibody (dilution: 1:200, Supplier: ABclonal, Cat: A7219)
Rabbit anti-ALKBH8 antibody (dilution: 1:200, Supplier: ABclonal, Cat: A7142)
Mouse anti-P53 antibody (dilution: 1:200, Supplier: Servicebio, Cat: GB13029-3)
Rabbit anti-ELP1 antibody (dilution: 1:200, Supplier: Abcam, Cat: ab115223)
Rabbit anti-ELP2 antibody (dilution: 1:200, Supplier: Abcam, Cat: ab154643)
Rabbit anti-ELP3 antibody (dilution: 1:200, Supplier: Abcam, Cat: ab113228)
Rabbit anti-ELP4 antibody (dilution: 1:200, Supplier: Abcam, Cat: ab133687)
Rabbit anti-CTU1 antibody (dilution: 1:50, Supplier: Abcam, Cat: ab185473)
Rabbit anti-CTU2 antibody (dilution: 1:200, Supplier: Abcam, Cat: ab177160)
Rabbit anti-ELP6 antibody (dilution: 1:200, Supplier: NOVUS, Cat: NBP1-91733)

Validation

When manufacturer validation was doubtful, antibodies were tested on positive controls and/or positive controls.

Eukaryotic cell lines

Policy information about cell lines

Cell line source(s)

Human GBC cell lines GBC-SD, SGC-996 and EH-GB1 were purchased from Cell Bank of Type Culture Collection of Chinese Academy of Sciences, and NOZ was obtained from the Health Science Research Resources Bank (Osaka, Japan). Human embryonic kidney 293T (HEK293T) cells were purchased from the American Type Culture Collection.

Authentication

All cell lines are commercial and authenticated.

Mycoplasma contamination

All cell lines were tested to be mycoplasma negative.

Commonly misidentified lines
(See ICLAC register)

No cell lines used in this study were found in the database of commonly misidentified cell lines that is maintained by ICLAC and NCBI Biosample.

Animals and other organisms

Policy information about studies involving animals; ARRIVE guidelines recommended for reporting animal research

Laboratory animals

Male BALB/c athymic nude mice, aged 4 weeks, purchased from the Shanghai Laboratory Animal Research Center (Shanghai, China).

this study.

Wild animals

No wild animals were used in this study.

Field-collected samples

No field-collected samples were used in this study.

Ethics oversight

All animal experiments were performed in accordance with relevant institutional and national guidelines and regulations.

Note that full information on the approval of the study protocol must also be provided in the manuscript.

Human research participants

Policy information about studies involving human research participants

Population characteristics

Surgically removed GBC tissue samples were obtained from individuals who underwent radical cholecystectomy at Renji Hospital, Shanghai Jiao Tong University School of Medicine between January 2008 and December 2014. GBC was diagnosed by histopathological examination.

Recruitment	Demography characteristics and clinical information of individuals were obtained from medical records. All the individuals underwent radical cholecystectomy received no chemotherapy or radiotherapy before surgery, and all individuals received gemcitabine-cisplatin chemotherapy after surgery. The bio-specimens from each individual were collected at the time of surgery.
Ethics oversight	This study was approved by the Ethics Committees of Renji Hospital, Shanghai Jiao Tong University School of Medicine .

Note that full information on the approval of the study protocol must also be provided in the manuscript.

Flow Cytometry

Plots

Confirm that:

- [x] The axis labels state the marker and fluorochrome used (e.g. CD4-FITC).
- [x] The axis scales are clearly visible. Include numbers along axes only for bottom left plot of group (a 'group' is an analysis of identical markers).
- [x] All plots are contour plots with outliers or pseudocolor plots.
- [x] A numerical value for number of cells or percentage (with statistics) is provided.

Methodology

Sample preparation	For apoptotic assays, cells were treated with gemictabine or vehicle. after indicated times, all cells were collected by trypsinization without EDTA,and 1 × 106 cells were doubly stained with Annexin V-FITC/PI
Instrument	FACS Calibur2Lasers
Software	FlowJo
Cell population abundance	No sorting was performed
Gating strategy	Gating strategy is provided in the manuscript.

- [x] Tick this box to confirm that a figure exemplifying the gating strategy is provided in the Supplementary Information.
